# Selectively UV-Blocking and Visibly Transparent Adhesive Films Embedded with TiO_2_/PMMA Hybrid Nanoparticles for Displays

**DOI:** 10.3390/ma13225273

**Published:** 2020-11-21

**Authors:** Jin-Wook Choi, Jun Hyup Lee

**Affiliations:** Department of Chemical Engineering, Soongsil University, Seoul 06978, Korea; cjw10546@naver.com

**Keywords:** display, hybrid nanoparticle, optically clear adhesive, transparency, UV protection

## Abstract

To simultaneously achieve the high visible transparency and enhance the ultraviolet (UV)-blocking performance of displays, inorganic–organic hybrid nanoparticles, comprising TiO_2_ as a core and poly(methyl methacrylate) (PMMA) as a shell, were uniformly incorporated into the optically clear adhesive (OCA) used in the front of a display device. The highly refractive TiO_2_ nanocore could selectively scatter UV rays, which degrade the display performance, owing to the differences in the refractive indices between the inorganic particles and PMMA matrix, thereby offering an improved UV protection property to the adhesive film. Moreover, the organic PMMA nanoshell maintained the high visible light transmittance of the pristine OCA film via the prevention of particle agglomeration. To examine the effect of the PMMA nanoshell and nanoparticle size on the optical properties of the adhesive films, the OCA films embedded with only TiO_2_ nanoparticles or hybrid nanoparticles with different particle sizes were prepared using a roll-to-roll process, and characterized in the range of UV and visible lights using UV-visible spectroscopy. It is experimentally revealed that the adhesive film including small TiO_2_/PMMA hybrid nanoparticles at an extremely low content exhibited enhanced UV-blocking properties and increased visible light transmittance compared to that with only TiO_2_ nanoparticles.

## 1. Introduction

As the ozone layer is being destroyed by environmental pollution, the amount of UV rays that directly hit the surface of the Earth is increasing. In addition, as the UV rays generated by the sun are detrimental to human skin and also adversely affect mobile phone displays, which are indispensable in modern daily life, many studies have been conducted with the objective of developing optical materials that block UV rays. When exposed to UV light for a long period of time, the color of the image displayed on a mobile phone display becomes altered or appears faded, and over time, the organic components of the display are degraded, which interferes with the normal operation of the device [1,2]. The UV rays that have such an adverse effect are largely divided into ultraviolet A (UVA), ultraviolet B (UVB) and ultraviolet C (UVC). Among these, UVA, which accounts for the largest proportion of the incident sunlight, penetrates glass at a wavelength of 320–400 nm and accounts for 95% of the radiation that reaches human skin. In addition, the blue light (UVA) that is generated within the display and directly hits human eyes has a negative effect on eyesight, and it must therefore be completely blocked [3,4,5]. The radiation wavelength of UVB is 290–320 nm, and it causes a degradation in the performance of the internal components of the display, thus shortening its life. Fortunately, the majority of UVCs are absorbed by O_2_ molecules, and therefore displays are primarily damaged by UVA and UVB rays. Therefore, if the display is not exposed to these wavelength bands, it can be protected, and its life can be extended [6].

One of the representative materials that blocks UV rays is TiO_2_ [7]. These particles are primarily used in the semiconductor field because their band gap is large, and thus their corrosion resistance to light is stable. They also have the advantage of being able to absorb UV light and light of lower wavelengths. The nanosized TiO_2_ particles used for UV-blocking have low dispersibility, and thus their formulation is agglomerated inside the matrix and they become white when coated on a film, which results in a white clouding problem that lowers the visible light transmittance. Thus, organic–inorganic hybrid material is the potential candidate to overcome these drawbacks [8,9,10]. An organic–inorganic hybrid material is a mixture of two or more components of an organic material and an inorganic material; it maintains the advantages of the existing material and has excellent physical properties [11]. Poly(methyl methacrylate) (PMMA) is widely used in the optical field, owing to its excellent optical properties, and it can offer high light transmittance as its refractive index is similar to that of the optically clear adhesive (OCA) used in the front of mobile phone displays [12,13,14].

A transparent adhesive layer is required to facilitate the application of a touch screen onto a display panel, such as an organic light emitting diode or liquid crystal display. One of the representative materials used as this adhesive layer is OCA. This is a sticky and transparent viscoelastic material used for the internal bonding of small mobile products to large electronic products, and is primarily used for the internal bonding of touch screens. In addition, as the OCA has a refractive index of 1.5, which is similar to those of other layers, including glass substrate, it can be considered visually transparent because it allows light to pass through in a straight line and without distortion [15]. Therefore, as the OCA plays an important role in the display, research is being conducted on methods to improve its performance [12,13,14].

**Figure 1:**
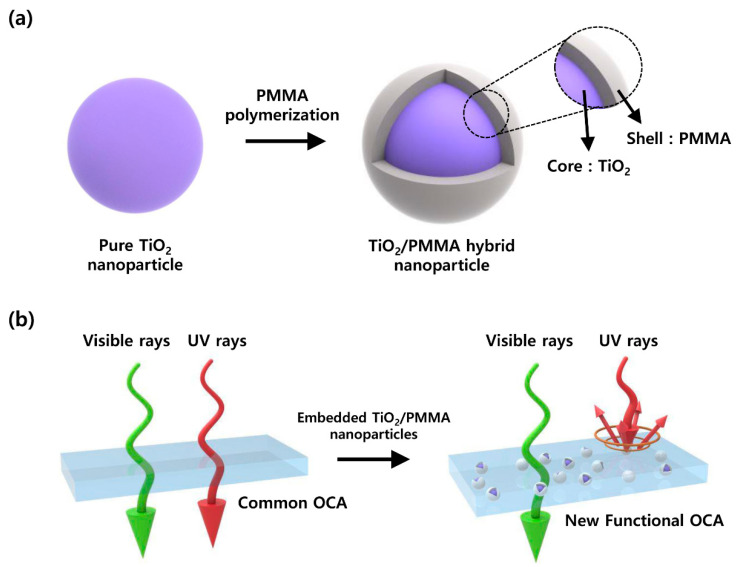
(**a**) Structure of inorganic–organic TiO_2_/PMMA hybrid nanoparticles. (**b**) Schematic of selectively UV-blocking and visibly transparent adhesive films.

In this study, a new type of functional optical adhesive embedded with TiO_2_/PMMA hybrid nanoparticles was proposed to simultaneously provide high visible light transmittance and an improved UV-blocking performance for displays (Figure 1). The inorganic–organic hybrid nanoparticles, comprising TiO_2_ nanoparticles as an the inorganic core and PMMA polymer as an organic shell, were synthesized via seeded dispersion polymerization. The organic nanoshell structure of the hybrid nanoparticle can improve the low dispersibility of inorganic TiO_2_ nanoparticles in the organic polymer matrix, due to its high compatibility with acrylic matrix polymers in the OCA film [16], thereby facilitating the high visible light transmittance of the film. In addition, as the TiO_2_ material has a band gap of 3.0–3.2 eV in the UVA area, it can absorb and scatter UV light, and thus protect the inner organic components of the display [17,18,19]. Moreover, the highly refractive TiO_2_ nanocore with a refractive index (n) of 2.6 can selectively scatter UV rays in low-refractive index acrylic polymers (n = 1.5), leading to the enhanced UV protection performance of the adhesive film [20,21,22,23]. To investigate the effects of the PMMA shell and the particle sizes of the hybrid nanoparticles on the optical properties of the OCA films, the nanoparticle-embedded optical films were fabricated using the roll-to-roll process, and their optical properties were characterized in the UV and visible regions.

## 2. Materials and Methods

### 2.1. Materials

TiO_2_ nanoparticles (21 nm, Aeroxide P25; and 300 nm, CR-50) were received from Cosmax (Seongnam, Korea). Since the particle size of the TiO_2_ nanoparticles is smaller than the wavelength of visible light, their optical transmittance is expected to be high. Methyl methacrylate (MMA) was purchased from Daejung Chemicals (Siheung, Korea). The MMA monomer was purified three times with NaOH and water to eliminate the radical inhibitor. The hydrogen chloride (HCl, 36.5 wt. %) used for the pH control of the TiO_2_ was received from Ducksan pure chemical (Ansan, Korea). Polyvinylpyrrolidone (PVP, MW = 29,000) as a dispersion stabilizer was purchased from Sigma Aldrich (Seoul, Korea). Methacrylic acid (MAA) was obtained from Junsei Chemicals (Tokyo, Japan) to modify the surface hydrophobicity of the TiO_2_. 2,2-Azobisisobutyronitrile (AIBN) as the radical initiator was received from Junsei Chemicals (Tokyo, Japan) and used after the purification. The UV-curable acrylic OCA was obtained from TMS company (Ilsan, Korea).

### 2.2. Synthesis of TiO_2_/PMMA Hybrid Nanoparticles

First, MMA and MAA were purified with NaOH and methanol, respectively, to remove the polymerization inhibitors. MAA (1 g), PVP (3 g) and methanol (300 mL) were placed in a circular flask and stirred at 25 °C and 1000 rpm for 10 min. After preparing an HCl aqueous solution (20 mL) with a pH of 2 in another beaker, 21 nm TiO_2_ (3 g) was dispersed at 1000 rpm for 30 min. The two dispersed solutions were mixed, and then AIBN (0.3 g) was added to the mixture, followed by heating to 60 °C and stirring uniformly. At 60 °C, the MMA (12 g) was added dropwise, and the reaction was performed at 300 rpm for 12 h. TiO_2_/PMMA hybrid nanoparticles were purified by centrifugation at 2500 rpm for 30 min and washing with methanol at 2500 rpm for 30 min three times [24,25]. Finally, to evaporate the solvent, it was dried in a vacuum oven at 25 °C for 24 h. By applying the same method to the 300 nm TiO_2_, TiO_2_/PMMA hybrid nanoparticles with a different particle size can be obtained.

### 2.3. Fabrication of the OCA Films Embedded with TiO_2_/PMMA Hybrid Nanoparticles

First, the pure TiO_2_ nanoparticles or the synthesized hybrid nanoparticles were added to an OCA solution and mixed well using a paste mixer (Thinky, AR-100, Tokyo, Japan) at 2000 rpm for 30 min. To prepare a thin film with a thickness of 200 μm, the OCA mixture was applied on a 75 μm thick release film, which was covered with a 50 μm thick release film, and the two release films were bonded using a roll-to-roll coater (MSRTR, SS-1, Seoul, Korea). To cure this bonded film, a UV curing machine (KJUV, KJPHT-101, Incheon, Korea) was used with a UV energy of 4 J cm^−2^ and at a UV intensity of 5 mW cm^−2^. To evaluate the optical properties of the cured film, the OCA film was cut to a size of 30 mm × 30 mm, and the UV–visible transmittance was measured.

### 2.4. Characterization

The zeta-sizer (Malvern Instruments, Nano ZS, Malvern, UK) equipped with dynamic light scattering was used to measure the size of the TiO_2_/PMMA hybrid nanoparticles. The chemical structure of the synthesized nanoparticles was determined by measuring the characteristic infrared (IR) peaks of the nanoparticles before and after the synthesis with a Fourier-transform IR spectrometer (FT-IR, Jasco, FT-IR 460 Plus, Tokyo, Japan) equipped with an attenuated total reflection (ATR) apparatus. To confirm the surface structure and morphology, the nanoparticles were measured at different magnifications after pretreatment with carbon tape and a platinum coating using a field-emission scanning electron microscope (FE-SEM, Hitachi, SU-70, Tokyo, Japan). Energy dispersive X-ray spectroscopy (EDS, Horiba, Energy X-MaxN, Tokyo, Japan) was used to determine the content of the elements in the nanoparticles. UV–visible spectroscopy (Scinco, S-4100, Seoul, Korea) was performed to measure the optical transmittance of the fabricated OCA films in the UV and visible regions.

## 3. Results and Discussion

### 3.1. Particle Size Characteristics of TiO_2_/PMMA Hybrid Nanoparticles

**Figure 2:**
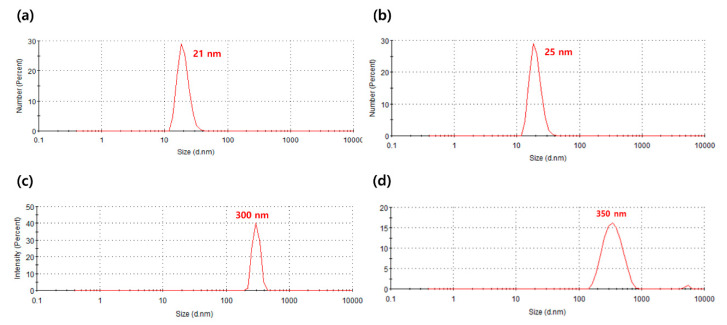
Particle size distribution curves of the pure TiO_2_ nanoparticles ((**a**) and (**c**)) and the synthesized TiO_2_/PMMA hybrid nanoparticles ((**b**) and (**d**)) with different particle sizes.

To compare the UV-blocking rate and visible light transmittance according to the size of the hybrid nanoparticles, the sizes of the nanoparticles were first measured. Figure 2 presents the particle size distribution curves of the pure TiO_2_ nanoparticles and the synthesized TiO_2_/PMMA hybrid nanoparticles with different particle sizes, using the dynamic light scattering of zeta-sizer. For measurement, the nanoparticles were dispersed in the deionized water with a concentration of 0.01 wt.%, and then placed in a transparent quartz cell. The received pure TiO_2_ nanoparticles showed uniform particle distributions with average particle sizes of 21 and 300 nm. On the contrary, the TiO_2_/PMMA hybrid nanoparticles exhibited increased particle sizes after the seeded dispersion polymerization of the MMA monomer. The pristine TiO_2_ nanoparticles of 21 nm size became TiO_2_/PMMA hybrid nanoparticles of 25 nm as the PMMA shell was formed on their core surface, and the TiO_2_ nanoparticles of 300 nm size changed to the hybrid nanoparticles of 350 nm. The increased particle sizes of the TiO_2_ nanoparticles, of 21 and 300 nm, were averagely 4 and 50 nm, respectively. Considering the particle size of the TiO_2_ core, the average thickness of the PMMA shell for TiO_2_ cores with 21 and 300 nm was approximately 2 and 25 nm, respectively, indicating the formation of an organic PMMA nanoshell on the inorganic TiO_2_ core. Therefore, it was confirmed that an ultrathin nanoshell of PMMA, corresponding to approximately 10% of the TiO_2_’s diameter, was stably formed on the surface of the TiO_2_ nanocore. The characteristics of each nanoparticle are summarized in Table 1.

### 3.2. Structural Characteristics of TiO_2_/PMMA Hybrid Nanoparticles

**Figure 3:**
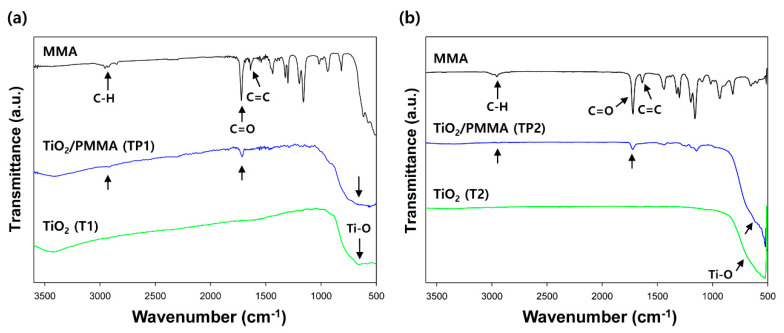
FT-IR spectra of the MMA monomer, the pure TiO_2_ nanoparticles ((**a**) and (**b**)), and the TiO_2_/PMMA hybrid nanoparticles ((**a**) and (**b**)).

The polymerization of the PMMA nanoshell on the seeded TiO_2_ nanoparticle was structurally analyzed using FT-IR spectroscopy. Figure 3 shows the FT-IR spectra of the MMA monomer, the pure TiO_2_ nanoparticles, and the TiO_2_/PMMA hybrid nanoparticles. First, the characteristic stretching vibration of the Ti–O bond at 650 cm^−1^ was clearly observed in the spectrum of the pure TiO_2_ nanoparticle. In the spectrum of the MMA monomer, the characteristic C–H and C=O stretching vibrations were detected at 2950 and 1735 cm^−1^, respectively. In addition, the stretching peak corresponding to the C=C double bond of the acrylate group was found at 1670 cm^−1^. After the seeded dispersion polymerization, the carbon–carbon double bond peak of MMA disappeared, but the characteristic vibrations of the PMMA polymer and TiO_2_ core were maintained in the spectrum of the TiO_2_/PMMA hybrid nanoparticle [26,27,28]. These results indicated that the PMMA was successfully polymerized on the surface of the TiO_2_ seed nanoparticles, and the inorganic–organic hybrid structure, comprising an inorganic TiO_2_ nanocore and an organic PMMA nanoshell, was constructed via the dispersion polymerization.

### 3.3. Morphological and Elemental Characteristics of TiO_2_/PMMA Hybrid Nanoparticles

**Figure 4:**
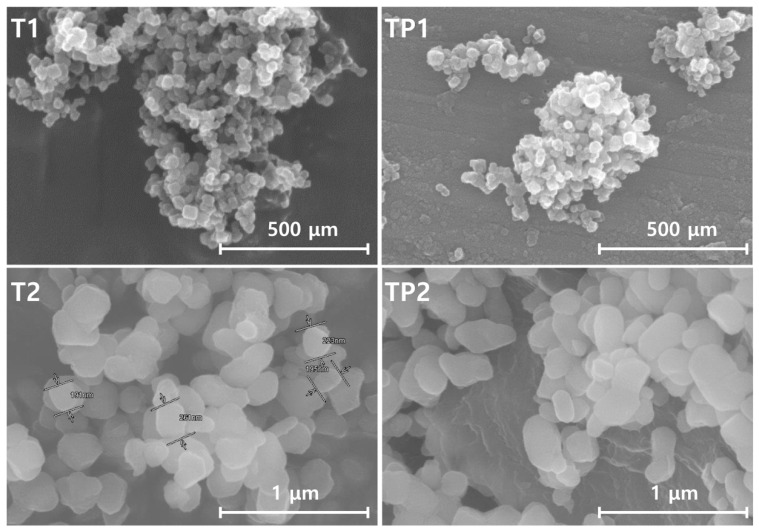
FE-SEM images of the pure TiO_2_ nanoparticles (T1 and T2) and the TiO_2_/PMMA hybrid nanoparticles (TP1 and TP2).

To confirm the change in surface morphology of the TiO_2_/PMMA hybrid nanoparticles after polymerization, field-emission scanning electron microscopy was conducted. Figure 4 presents the FE-SEM images of the pure TiO_2_ nanoparticles (T1 and T2) and the synthesized TiO_2_/PMMA hybrid nanoparticles (TP1 and TP2) with different particle sizes. The hybrid nanoparticles exhibited similar particle shapes to those of pristine TiO_2_ nanoparticles, but the particle sizes of the hybrid nanoparticles were increased after the formation of the PMMA shell. These results suggest that the spherical shape of the pristine TiO_2_ nanoparticles was retained even after the surface modification of the PMMA polymer, regardless of the particle size of the pure TiO_2_ nanoparticle, and the TiO_2_/PMMA hybrid nanoparticles with an increased particle diameter were successfully prepared.

**Figure 5:**
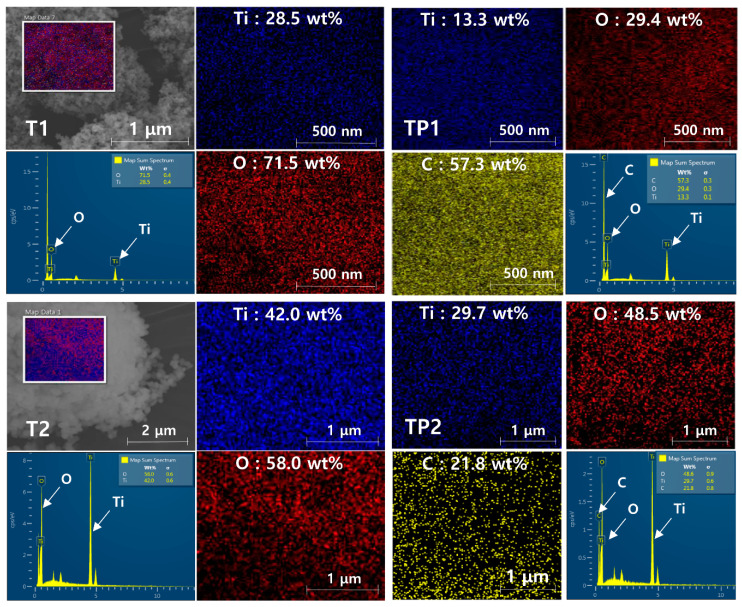
EDS mapping images and EDS spectra of the pure TiO_2_ nanoparticles (T1 and T2) and the TiO_2_/PMMA hybrid nanoparticles (TP1 and TP2).

Energy dispersive X-ray spectroscopy was performed to examine the elemental composition of the TiO_2_/PMMA hybrid nanoparticles. Figure 5 shows the EDS mapping images and EDS spectra of the pristine TiO_2_ nanoparticles and the TiO_2_/PMMA hybrid nanoparticles. Since the pure TiO_2_ nanoparticles are composed of titanium and oxygen atoms, the T1 and T2 nanoparticles exhibited the reasonable elemental composition of only Ti and O atoms. On the contrary, in the TiO_2_/PMMA hybrid nanoparticles (TP1 and TP2), a high content of carbon element was newly observed due to the formation of the outer PMMA shell layer. Therefore, the EDS mapping analyses revealed that the PMMA polymer as a nanoshell is successfully incorporated with the core of the TiO_2_ nanoparticles, and can construct the inorganic–organic hybrid structure.

### 3.4. UV-Blocking Characteristics of the OCA Films Embedded with TiO_2_/PMMA Hybrid Nanoparticles

**Figure 6:**
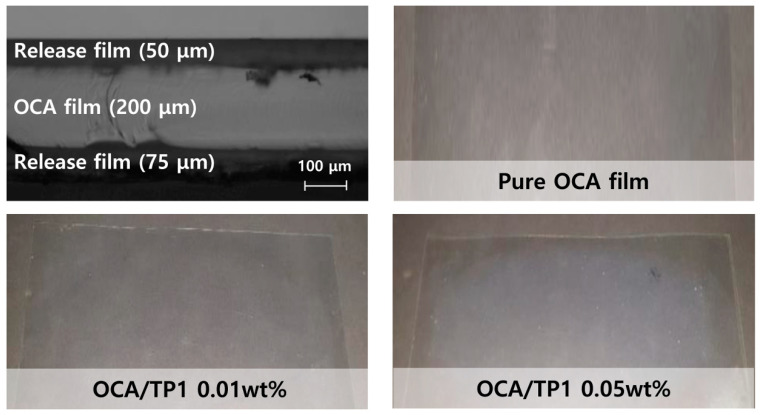
Optical microscopic and photographic images of the OCA films embedded with hybrid nanoparticles (TP1).

To measure the UV and visible light transmittance, the inorganic–organic TiO_2_/PMMA hybrid nanoparticles were mixed with the commercial OCA solution using a paste mixer, and then the nanoparticle-embedded OCA films with a thickness of 200 μm were fabricated between the two release films using a roll-to-roll coater. The concentrations of hybrid nanoparticles were 0.01 and 0.05 wt. %. As shown in Figure 6, the optical microscopic image of a cross section of the fabricated OCA film indicated the formation of a uniform thin film with a thickness of approximately 200 μm. Moreover, a large-area transparent film, as large as 624 cm^2^, was successfully prepared after the UV curing process.

**Figure 7:**
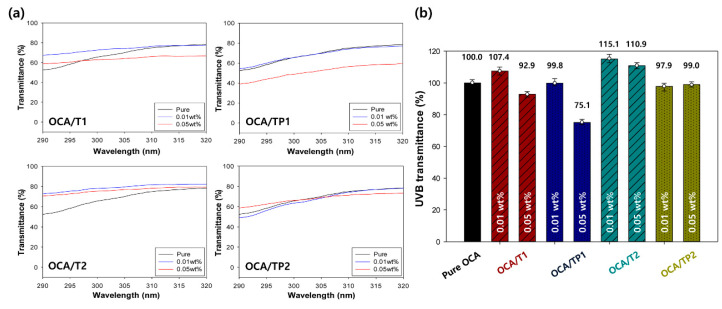
(**a**) UV–visible spectra and (**b**) average relative transmittances of the pure OCA and the nanoparticle-embedded OCA films in the region of UVB (290–320 nm).

With the reference value of 100% for a film fabricated from pure OCA, the relative optical transmittances of the nanoparticle-embedded OCA films were obtained in the UV and visible regions. UV rays can be absorbed or scattered by the TiO_2_ cores of hybrid nanoparticles owing to the presence of strongly bound excitons and high-refractive index characteristic. Figure 7a presents the UV–visible spectra of the pure OCA and the nanoparticle-embedded OCA films in the region of UVB (290–320 nm), according to the particle size and the concentration of the TiO_2_/PMMA hybrid nanoparticles. The average relative transmittance of each film at a UVB wavelength of 290–320 nm is summarized in Figure 7b. The OCA films with inorganic–organic TiO_2_/PMMA hybrid nanoparticles (OCA/TP1 and OCA/TP2) exhibited lower UVB transmittances than those with only pure TiO_2_ nanoparticles (OCA/T1 and OCA/T2), indicating an increase in the UVB-blocking performance compared to that of the film with common inorganic nanoparticles. In addition, a higher concentration and a smaller particle size of the hybrid nanoparticles showed better UVB light protection, and the maximum UVB-blocking rate of about 25% was achieved for the OCA film with TP1 hybrid nanoparticles at 0.05 wt.

% (OCA/TP1). Notably, even a small number of TP1 hybrid nanoparticles (0.05 wt. %) can provide an enhanced UVB protection ability to the OCA film.

**Figure 8:**
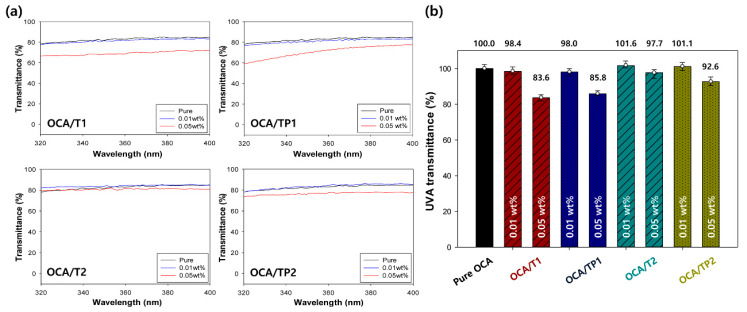
(**a**) UV–visible spectra and (**b**) average relative transmittances of the pure OCA and the nanoparticle-embedded OCA films in the region of UVA (320–400 nm).

Figure 8 shows the UV–visible spectra and average relative transmittances of the pure OCA and the nanoparticle-embedded OCA films in the region of UVA (320–400 nm) [29]. Similar results to those of the UVB region were obtained for the UV–visible spectra of UVA area, but the improvement rate in UVA protection performance was somewhat diminished, probably due to the increase in the wavelength of incident UV light relative to the particle size of the nanoparticles. The OCA film with TP1 hybrid nanoparticles (0.05 wt. %) exhibited the improved UVA-blocking rate of about 14%. From these results, it is concluded that the synthesized inorganic–organic TiO_2_/PMMA hybrid nanoparticles can afford an enhanced UV protection performance in the range of UVA and UVB, compared to that of common inorganic TiO_2_ nanoparticles, and the incorporation of small hybrid nanoparticles (TP1) into the OCA film at a relatively high concentration (0.05 wt. %) leads to the maximized UV-blocking rate of the film.

### 3.5. Visible Light Transmittance Characteristics of the OCA Films Embedded with TiO_2_/PMMA Hybrid Nanoparticles

**Figure 9:**
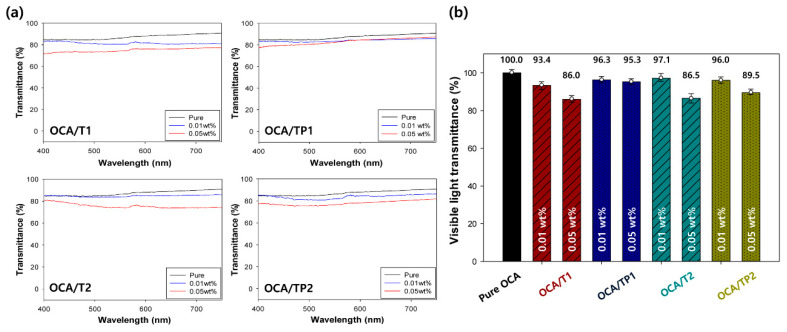
(**a**) UV–visible spectra and (**b**) average relative transmittances of the pure OCA and the nanoparticle-embedded OCA films in the visible light region.

Figure 9a presents the UV–visible spectra of the pure OCA and the nanoparticle-embedded OCA films in the region of visible light (400–750 nm) [30]. In addition, the average relative visible light transmittances based on the transmittance of the pure OCA film are shown in Figure 9b. The high visible light transmittance of the film is an essential prerequisite for the realization of a clear image without distortion in the display devices. In contrast to the UV transmittance, the visible light transmittances of the OCA films, including the TiO_2_/PMMA hybrid nanoparticles, were, on average, higher than those of the films with common inorganic TiO_2_ nanoparticles. This result is attributed to the increase in dispersibility of the hybrid nanoparticles in the OCA matrix, stemming from the surface modification of the organic PMMA polymer on the inorganic TiO_2_ core [31]. Furthermore, a lower concentration and a smaller particle size of hybrid nanoparticles led to the higher visible light transmittance of the film. Notably, even a high concentration of TP1 hybrid nanoparticles (0.05 wt. %) provided higher visible light transmittance (95.3%) comparable to that of a low-concentration hybrid nanoparticle (96.3%), indicating the excellent dispersibility of small hybrid nanoparticles. As a consequence, it is concluded that when the visible light transmittance and UV-blocking rate of the fabricated OCA films were compared, the OCA film fabricated with small TP1 hybrid nanoparticles at a relatively high content of 0.05 wt. % demonstrated the best UV light-blocking ability and visible light transmittance.

## 4. Conclusions

The inorganic–organic TiO_2_/PMMA hybrid nanoparticles were employed as a functional optical filler of the OCA film to selectively block the UV light and eliminate the particle agglomeration of common inorganic TiO_2_ nanoparticles, due to their poor dispersion in the matrix, resulting in the fabrication of a visibly transparent adhesive film with an improved UV-blocking performance for displays. The synthesized TiO_2_/PMMA hybrid nanoparticles with different particle sizes were successfully characterized via zeta-sizer, FT-IR, FE-SEM, and EDS, and mixed with the OCA solution to form a thin film. On measuring the visible light transmittance and UV light-blocking rate according to the particle size and content of the hybrid nanoparticles, the OCA film including small TP1 hybrid nanoparticles with an average diameter of 25 nm demonstrated approximately a 25% UVB-blocking rate, a 14% UVA-blocking rate, and 95% visible light transmittance at a relatively high content of 0.05 wt. %. Based on these results, it was concluded that the TP1 hybrid nanoparticle exhibited the best optical performance by selectively blocking UV light and transmitting visible light. When these inorganic–organic hybrid nanoparticles are added to the OCA film, which is an optical adhesive used inside a display, it can effectively block harmful UV wavelengths and prevent the particles from becoming cloudy, thereby making them suitable for application in the medical and beauty industries, in addition to the optical industry.

## Figures and Tables

**Table 1:** Characteristics of the pure TiO_2_ nanoparticles and the TiO_2_/PMMA hybrid nanoparticles.

No.	Code	Material	Average Particle Size	Surface Characteristic
1	T1	TiO_2_	21 ± 2.7 nm	Inorganic
2	TP1	TiO_2_/PMMA	25 ± 3.3 nm	Organic
3	T2	TiO_2_	300 ± 23.9 nm	Inorganic
4	TP2	TiO_2_/PMMA	350 ± 93.2 nm	Organic
